# Recent Drugs and Remedies

**Published:** 1887-10-01

**Authors:** 


### RECENT DRUGS AND REMEDIES.

By our Special Commissioner.

### ELECTRICITY.

Those who anticipated a new era of startling cures on the introduction of electricity into therapeutics have been considerably disappointed. The miracles have not been wrought, and the wonders yet remain to be witnessed. The disappointment has reacted injuriously, and now many people have ceased to expect any practical results of decided value from electrical treatment. The exaggerated anticipations were not based on reason, and the present indifference is just as irrational. There can be no doubt that electricity has an important part to play in the medicine of the future. It can never be a substitute for all other methods of practice, a kind of "quack pill" which will cure everything, earthquakes included. But as an adjunct to other kinds of treatment it has a distinct field of its own, in which it cannot be supplanted.

No one who is the least familiar with the action of electrical stimulus upon nerve, muscle, and the blood and lymph streams, or with the history of physiological and therapeutic experiment from the time of Duchenne until now, will doubt the potency of the agent under consideration. Whatever is thought of its value, its power cannot be denied. But those who have carefully studied the history of recent experiments, and who have in addition had some practical experience of the subject, are convinced that in electricity, properly applied, there is the possibility, nay, the certainty of great and beneficial results to a portion of mankind. In certain cases of chronic rheumatism, and what is called "rheumatic gout," perhaps nothing else offers such reasonable prospect of good as electricity. Similarly, in a variety of nerve and muscle affections, the judicious and persevering use of the electric stimulus will often be productive of the most satisfactory results. Many cases of dyspepsia and languor, arising from a want of energy in the blood and lymph circulations, would probably yield to electricity more promptly and completely than to any other method of treatment.

At Southsea there may now be witnessed a new departure in this line of things. An "electrified room" is there in full operation, under the supervision of Mr. Wodsworth, M.R.C.S. The particular method of applying the treatment is the idea of Captain Arthur Byng, of the Royal Navy, and although it is protected by patent, there is nothing secret or mysterious about it. The room is electrified by means of an insulated floor connected with one pole of a coil, and an insulated ceiling similarly connected with the opposite pole. The patient, standing or seated between the two, is acted upon by induction. This is easily demonstrated by a very simple experiment. The subject placed on the insulated floor is charged with electricity, though he is not conscious of it; but let him now be touched by an attendant standing on the wooden floor of the room, and he immediately becomes conscious of the usual sensations accompanying the passage of the current; thus proving that the electricity passes from him, through the attendant, to the floor. The value of this for any person who cannot bear the ordinary shocks is obvious. Of course there are at the rooms all the usual appliances for administering the treatment locally, as well as electric baths. The medical profession in Portsmouth and Southsea have taken up the idea with much liberality of spirit, many of them sending patients for treatment, and some of them speaking very highly of the results. Sir William Aitken, of Netley, author of Aitken's "Practice of Medicine"; Surgeon-General Maclean, of Netley, and other eminent military surgeons, have visited the rooms, and expressed themselves as much pleased with the decided advance which they mark in the progress of the therapeutics of electricity. A room is shortly to be fitted up at the Netley Hospital for experimenting with those cases which are likely to be benefited, and which are by no means infrequent among officers and men in the army. In the meantime some very striking cases are reported as having been cured or greatly improved, and it is very desirable that medical men who have patients for whom electricity is practically the only hope left, should make themselves acquainted with the methods in use at the rooms. Dr. Wodsworth, a retired army surgeon of very conservative principles of medical etiquette and propriety, will give any information to medical men they may desire. The Company are anxious to avoid the methods of quackery, and therefore treat cases only through the regular professional channels.

